# A conceptual map of health-related quality of life dimensions: key lessons for a new instrument

**DOI:** 10.1007/s11136-019-02341-3

**Published:** 2019-11-01

**Authors:** Jan Abel Olsen, RoseAnne Misajon

**Affiliations:** 1 Department of Community Medicine, University of Tromsø - The Arctic University of Norway, Tromsø, 9037 Norway; 2 Norwegian Institute of Public Health, Oslo, Norway; 3 School of Public Health and Preventive Medicine, Monash University, Melbourne, Australia; 4 The Cairnmillar Institute, Melbourne, Australia

**Keywords:** QALYs, Health state utility instruments, EQ-5D, SF-6D

## Abstract

**Purpose:**

Quality-adjusted life years (QALYs) represent a critical metric in economic evaluations impacting key healthcare decisions in many countries. However, there is widespread disagreement as to which is the best of the health state utility (HSU) instruments that are designed to measure the Q in the QALY. Instruments differ in their descriptive systems as well as their valuation methodologies; that is, they simply measure different things. We propose a visual framework that can be utilized to make meaningful comparisons across HSU instruments.

**Methods:**

The framework expands on existing HRQoL models, by incorporating four distinctive continua, and by putting HRQoL within the broader notion of subjective well-being (SWB). Using this conceptual map, we locate the five most widely used HSU-instruments (EQ-5D, SF-6D, HUI, 15D, AQoL).

**Results:**

By individually mapping dimensions onto this visual framework, we provide a clear picture of the significant conceptual and operational differences between instruments. Moreover, the conceptual map demonstrates the varying extent to which each instrument moves outside the traditional biomedical focus of physical health, to also incorporate indicators of mental health and social well-being.

**Conclusion:**

Our visual comparison provides useful insights to assess the suitability of different instruments for particular purposes. Following on from this comparative analyses, we extract some important lessons for a new instrument that cover the domains of physical, mental and social aspects of health, i.e. it is in alignment with the seminal 1948 WHO definition of health.

## Introduction

The last few decades have seen a proliferation in the development of health-related quality of life (HRQoL) instruments. These instruments are commonly categorised along two important dichotomies. First, whether they are disease specific or generic, with the latter intended to enable comparisons of HRQoL across patients with *different* diseases. Second, whether their scoring system, or value sets, are based on individuals’ preferences. The sub-group of instruments that are both generic *and* preference based are referred to as generic preference-based measures (GPBM) [1]; health state utility (HSU) instruments [2], or; Multi Attribute Utility Instruments (MAUI) [3]. Given that these instruments are developed for the purpose of valuing *health* states, we opt for the most telling acronym HSU in the current paper.

Beyond being generic and preference based, a further characteristic of HSU-instruments is that their value sets enable comparisons of gains in terms of health state improvements with gains in terms of increased life expectancy. Thereby, HSU instruments serve the purpose of measuring and valuing the Q, or the *quality* part, in the QALY (quality-adjusted life years) which is a commensurable metric of health outcomes [4]. QALYs represent a crucial metric in economic evaluations and health technology assessments, which have come to influence healthcare decision making in many countries, e.g. NICE in the UK [5]. Hence, as compared to other HRQoL instruments, HSU instruments have gained a most dominant position in health policy-making.

Reviews of the literature have typically identified five (or sometimes six) HSU-instruments [1, 3, 6]. The EQ-5D is by far the most widely applied, followed by the SF-6D. Three instruments have mainly been applied within their countries of origin: the HUI in Canada, the 15D in Finland, and the AQoL in Australia. Finally, the QWB is the least applied, which is likely due to it being the longest instrument (71 items with an estimated 20 min to complete). Because of its peripheral position in the applied literature, the QWB will not be included for discussion in the current paper.

There is persistent disagreement in the literature on exactly which instrument is *the* best, or the least bad. Their descriptive systems differ in the following ways: (i) details and length, from the brief EQ-5D towards the long AQoL; (ii) the number of dimensions included, from 5 to 15; (iii) the breadth of the concept, from an emphasis on physical health (EQ-5D) to including mental health and social relationships, and even happiness (AQoL); (iv) whether the emphasis is on symptoms/causes (HUI) or functioning/effects (SF-6D). Hence, when respondents are being asked to value different things, and the valuation methodologies differ, there is no surprise the inferred health state utilities differ across instruments [2, 7].

The descriptive variation across HRQoL instruments reflects the gradual policy shift in recent years around notions of ‘health and well-being’ (see e.g. [8], with an increasing interest into the use of well-being measures to inform policy [9]. In addition, there has been rapid growth in the research area of subjective well-being (SWB) and related concepts such as ‘life satisfaction’ and ‘happiness’. These shifts have resulted in an intersection between the field of HRQoL and that of SWB [10], therefore, compelling researchers to better articulate how HSU instruments sit in relation to broader notions of health *and* well-being.

The aim of this paper is twofold. First, we develop a conceptual framework, illustrated by a figure, in which we locate the five HSU instruments. This is for the purpose of visualising their similarities and contrasts. As compared to previous comparisons of the domain contents in HSU instruments [1, 3], we extend the conceptual analysis beyond a simplistic physical versus psycho-social dichotomy, namely incorporating potential causal relationships and level of abstraction in the framework. A visual framework provides a unique way to represent these conceptual complexities and provides a potentially powerful tool to better understand how different instruments operationalise the concept of HRQoL. Second, following on from this conceptual map we extract some important lessons on which dimensions to include in a new instrument that is more aligned with the current pattern of health problems that healthcare, care and social services seek to improve.

The paper is structured as follows: The next section outlines four analytical distinctions before it leads on to the conceptual map which locates those dimensions that have been included in at least two of the five HSU instruments. Section Contrasts across HSU-instruments compares the key characteristics of each instrument with corresponding figures to illustrate where they belong in the general conceptual map. Furthermore, while the new PROMIS instrument is not classified as a HSU-instrument, the descriptive system of its shortest version will be briefly discussed in light of the fact that a preference based summary score is now being developed [11]. Section Lessons for a new descriptive system for Health Related Quality of Life draws some lessons on which dimensions to include when developing a new or revised instrument. The suggested dimensions cover the physical, mental and social domains of health, which are aligned with the seminal WHO definition of health from 1948. Finally, Sect. Discussion contains a discussion and concluding remarks.

## A conceptual framework of HRQoL dimensions

Our conceptual framework expands on two models that are widely used to explain the relationships between various dimensions of HRQoL. The first is the International Classification of Functioning, Disability, and Health (ICF) which is a WHO-endorsed detailed classification system providing a standard language and framework for the description of health and health-related states (http://www.who.int/classifications/icf/en/). The ICF consists of two parts: Part 1 refers to functioning and disability, and consists of two key components: Body functions and structures, and; Activities and participation. Body functions refer to physiological (and psychological) functions of the body systems, and hence, impairments are considered as problems in such body functions. Activities refer to the execution of a task or action, while participation refers to the involvement in life situations. HRQoL dimensions usually fall within this first part.

Part 2 of the ICF refers to contextual factors, and consists of *Environmental factors* (physical, social and attitudinal environment) and *Personal factors* (gender, age, habits, coping styles, education etc.). Social support and relationships are considered an environmental factor, but also included in the ‘Activities/Participation’ chapter of the ICF. There is an important difference as to whether social support and relationships are measured as *background* factors (that may impact on health outcomes), or whether they are measured as a *consequence* of health.

The second model, on which our conceptual framework is based, is the revised Wilson and Cleary model [12, 13]. This model articulates more clearly the relationship between HRQoL components, depicting dominant causal pathways between each of the five levels of health outcomes: Biological and physiological variables → Symptom status → Functional status → General health perceptions → Overall quality of life. The model acknowledges the roles of individual and environment characteristics to influence the last four of these levels.

Inspired by the above models, and by issues with HRQoL measurement raised by previous researchers [14–16], our proposed framework is built around four key distinctions, which, however, emerge more like continua than dichotomies: (1) cause versus effect; (2) specific versus abstract; (3) physical versus psychological, and; (4) subjective *vs* objective.

### Cause versus effect

Cause indicators are those that can result in a change to the end-state, while effect indictors are the measured end-state(s). Thus, for example, symptoms such as pain can be considered cause indicators that can impact on the capability to function, e.g. *mobility*. Next, when mobility impacts on *usual activities*, it takes an intermediate role in the causal chain. While the cause-effect continuum is aligned with the Wilson and Cleary model, the direction of the causal arrows is an issue of debate, e.g. dysfunction may cause depression (a symptom), and social isolation may have negative impacts on health [17]. While we acknowledge bidirectional links across some life domains and HRQoL-dimensions, still there is recent evidence that among EQ-5D items, pain and depression are causal, that impact on self-care and usual activities [18, 19], and that ill health have strong impacts on social relationships [20].

### Specific versus broad

Almost parallel to the [cause → effect] continuum is the [specific**—**broad] continuum. Generally, cause indicators (symptoms) tend to be specific (e.g. hearing, vision), while dimensions like *self*-*care* and *social functioning* become less specific. We then have the broadest items that require respondents to consider their overall well-being. In recognition that causal relationships are sometimes less clear due to being bidirectional, the [specific**—**broad] continuum serves as an important anchoring guide on the framework.

### Physical versus psychological/mental

A well-known dichotomy in the literature is that between physical *vs* psychological aspects of health. Generally, the more specific the item, the easier it is to classify it according to this distinction. Thus for instance, items that clearly reflect the ICF’s ‘Body structures’ or ‘Body functions’ classification would more readily be categorised according to this distinction. However, as the item measures something broader, the boundaries between physical and psychological become less clear. For example, activity limitations and participation restrictions can stem from *either* physical *or* psychological issues. Likewise, items tapping into social functioning or social relations may capture both physical and psychological components of health.

### Subjective versus objective

A key distinction within the field of quality of life research is that between subjective and objective variables. Objective variables are tangible and can be observed and measured by others. Subjective variables on the other hand, involve people rating their feelings or assessments (e.g. feeling blue) which cannot be verified by others as they are private to the individual making the assessment. Generally, variables tapping into physical health domains can be more readily verified by observation (e.g. ‘walking 5 m unaided’), in comparison to psychological domains that are often based on a patient’s self-report. However, pain is usually included in measurements of ‘physical health’ but is primarily dependent on self-report.

### ‘Science illustrated’: the conceptual framework set in a figure

**Fig. 1:**
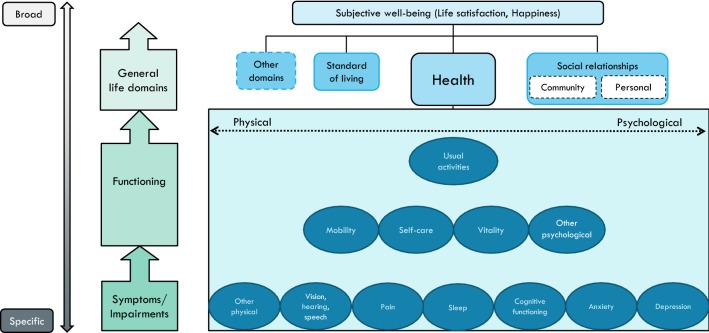
A conceptual map of health and well-being: The health dimensions included in HSU instruments, and the general life domains included in the PWI, placed in a theoretical context

These four distinctive continua form the basis for visually representing our conceptual framework in Fig. 1. On the vertical axis, the [specific**—**broad] continuum runs parallel to the causal continuum [symptoms → functioning → general life domains → well-being]. While the lower outcome levels are similar to the Wilson and Cleary model, our framework differs at the higher outcome levels in that it places HRQoL within the SWB framework.

Among the various questions and instruments used for measuring subjective well-being/happiness (see [21], the Personal Wellbeing Index (PWI) [22, 23] is particularly relevant in the current context. After an initial question on global life satisfaction, the PWI attends to general life domains by asking about the satisfaction with: standard of living; health; community connectedness; personal relationships; achievement; safety, and; future security. The first four of these domains have been referred to among items included in HSU-instruments, and are therefore included in Fig. 1. At the top level of the figure lies subjective well-being, or ‘overall quality of life’, that can best be defined as a combination of cognitive evaluation of one’s life (e.g. life satisfaction) and affect (e.g. happiness).

On the horizontal axis, we represent the physical–psychological distinction as a continuum rather than two distinct categories (under the domain of ‘health’), to reflect the difficulty of categorizing items into one over the other. It is less difficult to map the progression of physical health items from specific (symptoms, cause indicators) to broad (effect indicators, perceived end-state) than it is for psychological health items, due to the level of subjectivity and abstraction attached with the latter.

The initial design of the map included a [subjective**–**objective] continuum, running parallel to the [physical**–**psychological] continuum. This would locate items on the degree of being observable, or primarily dependent on patients’ self-reports. However, given that the nature of HSU-instruments is to collect participants’ *subjective* descriptions by rating the severity of the various dimensions, rather than using objective indicators of health as measured by clinicians, this distinction is not represented on the conceptual map.

Once completing the basic framework, the next step was to incorporate specific HRQoL dimensions. This is where we analysed the five HSU questionnaires, creating a table to categorise each item into specific dimensions (see Table 1). While based on slightly different categories, similar comparisons of instrument contents have been made by Brazier et al. [1] and Richardson et al. [3, 7].

**Table 1:** Comparisons of the dimensions included in the five HSU-instruments

	EQ-5D	SF-6 D^a^	HUI-3 (8 items)	15D	AQoL-8D (35 items)
Symptoms					
Vision			1	1	1
Hearing			1	1	1
Speech/communicate			1	1	1
Pain	1	1	1	1	2
Sleep				1	1
Cognitive functioning			1	1	
Anxiety	1	1		1	2
Depression				1	3
Other physical			1	3	
Functioning					
Mobility	1	1	1	1	2
Self-care	1	1			1
Vitality		1		1	1
Other psychological			1		10
Usual activities	1	2		1	1
Relationships and wellbeing					
Personal relationships					3
Community connectedness					4
Happiness/life satisfaction					1
*Unclassified:* intimacy/sexuality				1	1

^a^The current categorization of SF-6D is based on Brazier et al. [1]. Two different versions of the SF-6D are based on either 11 or 10 items from SF-36 (Brazier et al. [29]

To reduce the number of dimensions in Fig. 1, at least *two* of the five HSU-instruments would have to include an item which clearly reflect that dimension. Thus, the map includes the following HRQoL dimensions at the symptoms level: senses, sleep, pain, cognitive functioning, anxiety, depression, and an additional dimension ‘other physical’. Above, at the functioning level, we have mobility, self-care, vitality, and ‘other psychological’. Above, at the next functioning level, we locate usual activities. Then, at the level above, the general life domains are depicted, that in turn determine global life satisfaction at the top level. By use of this map, we can further compare and contrast the five HSU-instruments.

## Contrasts across HSU-instruments

By mapping the five instruments into the conceptual framework, their contrasting differences are visualised. Two instruments (HUI and 15D) emerge as symptoms oriented, while two other (EQ-5D and SF-6D) emerge as more functioning oriented. Finally, one instrument (AQoL) emerges as a hybrid of a health and well-being instrument that includes a broad spectrum of items on symptoms, functioning, social relationships and even happiness.

### Two symptoms-oriented instruments

**Fig. 2:**
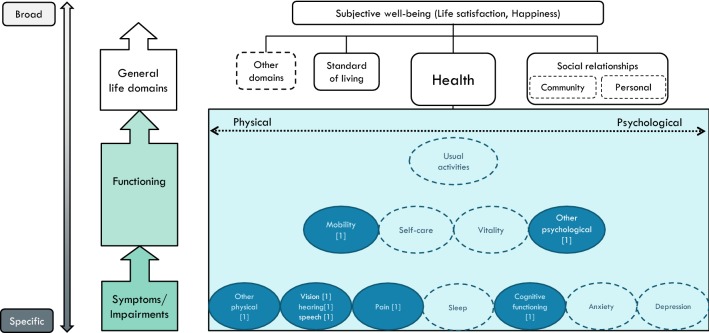
A conceptual map of the Health Utility Index, HUI (8 items). **a** Other psychological: happy/unhappy (emotions), **b** Other physical: dexterity

The *HUI3* is the third and latest version of the Health Utility Index (www.healthutilities.com) and includes eight items focusing heavily on symptoms (cause indicators), with the majority tapping into physical symptoms. Other than ‘cognitive functioning’, no items capture psychological health at the level of symptoms or activity. However, the *Emotion*-item describes different levels of ‘happiness’, which is the affect component of subjective well-being that tap into the highest level of abstraction and bypassing all levels in between. We chose the heading—or label—of this item (Emotion) to dictate its location in the map as ‘Other psychological’, see Fig. 2. Arguably, the inclusion of this dimension may seem at odds with the general focus of the *HUI3*.

**Fig. 3:**
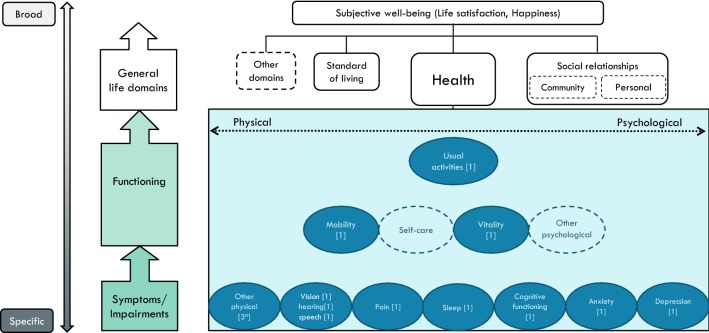
A conceptual map of the 15D (14 items mapped, excluding the sex item: impact of health on sexual behaviour). **a** Other physical: eating, breathing, elimination

The *15D*-instrument (http://15d-instrument.net/15d/) includes 15 items. It provides full coverage of both physical and psychological dimensions at the symptom level (cause indicators), plus three dimensions at the activity/participation level (effect indicators). Above that, it includes one item on the ability to perform ‘usual activities’. The instrument’s 15th item on ‘sexual activity’ was difficult to locate within our conceptual map, (given the role of physical, psychological and social aspects on sexual activity) and was, therefore, not included in Fig. 3.

### Two functioning-oriented instruments

**Fig. 4:**
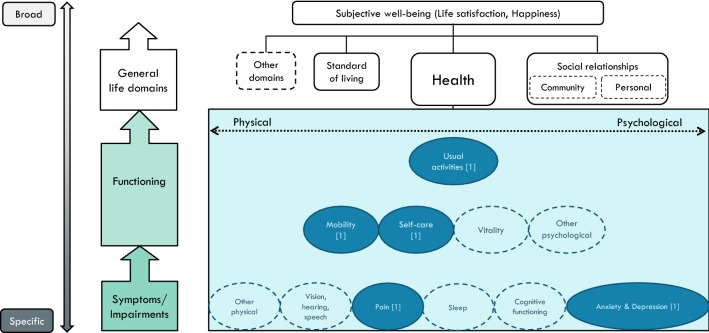
A conceptual map of the EQ-5D (5 items)

The *EQ*-*5D* (https://euroqol.org/) includes five dimensions/items: mobility, self-care, usual activities, pain/discomfort, anxiety/depression. In its original version, each dimension was described using three levels of severity (EQ-5D-3L), while the recent version uses five levels (EQ-5D-5L). The first three items have an emphasis on functioning, while the remaining two describes physical symptoms (pain) and psychological symptoms (an amalgamation of anxiety and depression), see Fig. 4.

**Fig. 5:**
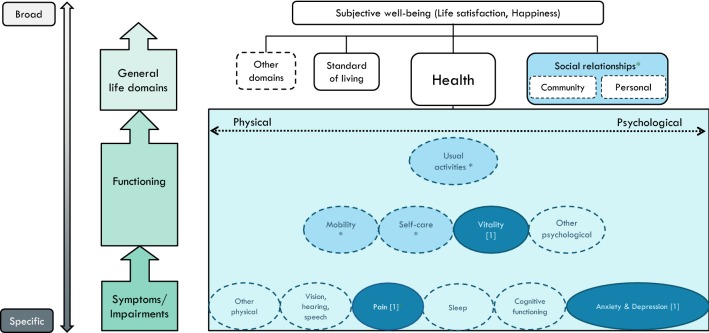
A conceptual map of the SF-6D. *The item Physical functioning relates to Mobility and Self care; the item Role functioning relates to Self-care and Usual activities; the item Social functioning refers to limitations in social activities, which relates to Social relationships; the item Mental health refers to Depression and ‘very nervous’ which relates to Anxiety

The *SF*-*6D* (https://www.sheffield.ac.uk/scharr/sections/heds/mvh/sf-6d) is derived from a selection of items in the SF-36. It includes six dimensions: physical functioning, role functioning, social functioning, pain, mental health, energy. Each dimension is described using five levels of severity (except ‘pain’ using six levels). Interestingly, in four of the six items, severity is described in terms of the frequency at which a problem occurs; from ‘none of the time’ to ‘all of the time’. Note also that the first three dimensions, in its wording, explicitly focus on ‘functioning’. As compared to the above three instruments, the SF-6D put more emphasis on psycho-social aspects of health. One such psycho-social item has a generic heading; ‘mental health’, but the description of its various severity levels refers more specifically to being ‘depressed or very nervous’ at different frequencies (Fig. 5).

### One hybrid instrument

**Fig. 6:**
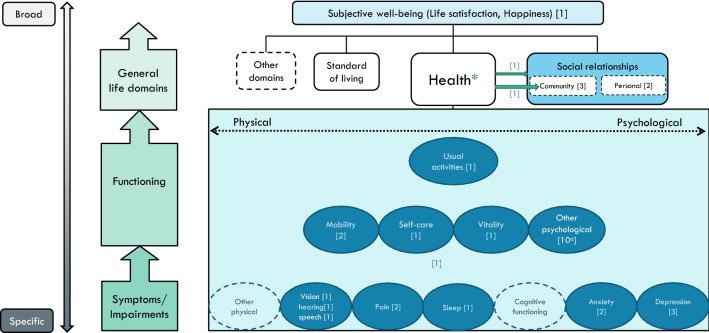
A conceptual map of AQoL-8D. ^a^Other psychological: dependence, control, coping, anger, confidence, self-worth, self-harm, happy (× 2)

As compared with the other HSU-instruments, the *AQoL* has a broader scope emphasising health *and well-being*. The *AQoL* (https://www.monash.edu/business/che/aqol) comes with four different versions: The longest (8D) includes 35 items divided between eight dimensions: Independent Living, Happiness, Mental Health, Coping, Relationships, Self-Worth, Pain, Senses. The shortest (4D) includes 12 items split between four dimensions: Independent Living, Mental Health, Relationships, Senses. The longest version is mapped in Fig. 6, and shows its focus on psychological aspects with items measuring ‘other psychological’, and multiple items tapping into anxiety and depression. At the top end of the [specific**—**broad] continuum, AQoL includes items measuring global life satisfaction and happiness. It also includes several items asking about social relationships, plus the *impact* that health has on social relationships.

### One new contender, however, not considered a ‘health state *utility*’ instrument

The *PROMIS* (http://www.healthmeasures.net/index.php) includes a range of instruments for use with the general population and with individuals living with chronic conditions. The PROMIS contains an extensive item bank, structured in a hierarchy illustrated with three headings: Physical Health, Mental Health, Social Health, something that gives a strong resemblance to the seminal WHO definition of health.

Note that the value sets, or scoring systems, on which the PROMIS instruments are based, do not apply preference based methodologies. Thus, PROMIS is not being classified in the literature as a generic *preference* based measure (GPMB), nor a health state *utility* (HSU) instrument. However, work is currently being done on selecting key health domains in a brief descriptive system, for which preference-based summary scores can be developed. The questionnaire contains seven key domains each of which are presented with two items: Physical function; Depression; Pain; Fatigue; Cognition; Social roles, and; Sleep [11, 24].

## Lessons for a new descriptive system for Health-Related Quality of Life

The above conceptual comparison provides important lessons for developing an improved descriptive system. A first lesson is to acknowledge the vast research efforts in the process of selecting those dimensions that are contained in existing instruments. Thus, the most common denominators are the strongest candidates for continued inclusion: All instruments contain items on *pain* although its descriptions differ, emphasising either symptoms or functioning aspects of pain. All instruments contain items on *mobility*, or described as ‘physical functioning’ in SF-6D and PROMIS. All instruments include *depression*, except for the HUI which uses the term ‘emotion’, however, described along different levels of ‘happiness’—a word that arguably brings connotations to (absence of) depression.

A second lesson is that a brief descriptive system appears to be necessary to have it included in applied studies. Note that the three shortest HSU-instruments (EQ-5D, SF-6D; HUI) are used in around 90% of published cost-utility analyses, leaving the three long ones (QWB, AQoL, 15D) for the remaining 10% [6, 25]. Brevity of an instrument not only refers to its number of items, but also that the words used should be understandable without any need for further explanations of their meaning.

A third lesson is to acknowledge the breadth of the concept of health, cf. the seminal WHO definition which has not been amended since 1948: “Health is a state of complete *physical, mental* and *social* well-being and not merely the absence of disease or infirmity”. With this in mind, note that the most recently developed generic descriptive system, PROMIS, is built around the domain headings of physical, mental and social health.

A further consideration is to develop a descriptive system that is aligned with the changing pattern of health problems that current resources on healthcare, care and social services seek to improve. Over the last decade, much policy attention has been directed towards mental health and social isolation. The evidence that mental health is crucial for health-related quality of life implies that it should be described using at least two, preferably three (potentially four) items. Given that impairments in physical and mental health reduce our social functioning, and the evidence that social relationships are the most important determinant of subjective well-being [26], and particularly so among the older people [27], (as well as acknowledging its policy attention to the extent that the UK prime minister Theresa May appointed a minister for loneliness in a drive to tackle social isolation), two social health dimensions should be included that attend to: people’s relationships with their inner circle of friends and family, and; their connectedness to the wider community.

Social aspects of health are completely ignored in the EQ-5D, the HUI and the 15D. While accounted for in the SF-6D, it has an even more central place in the AQoL and the PROMIS. Furthermore, when moving from health dimensions to the general life domains described by the PWI, we note that two of its seven domains refer to social relationships.

The suggested new descriptive system would contain eight (possibly nine) dimensions in total, set under three general health domains: The *physical health domain* includes three dimensions; mobility, self-care, pain. As for self-care, this dimension is included in four instruments, but not in the two symptoms oriented ones (HUI and 15D).

The *mental health domain* would include three (possibly four) dimensions: *depression, vitality, sleep* (and possibly *anxiety*). Note that in both the EQ-5D and the SF-6D the description of ‘depression’ go wider, to include ‘anxiety’ in the EQ-5D or ‘very nervous’ in the SF-6D. There are, however, good clinical reasons to separate out ‘anxiety’ in a stand-alone dimension, but it remains an empirical question whether feelings of anxiety would alternatively be picked up by the other three mental health dimensions. Vitality, which is present in four instruments, represents an important aspect of mental functioning, and should therefore be included. Sleeping problems can be *caused* by anxiety-type feelings of nervousness, worries or stress, and have important *effects* on overall functioning. Furthermore, given that ‘sleep’ is a word that people can easily understand and rate, there are good reasons to include it as a separate item. Certainly, much work is required to determine which mental health dimension to include, as well as finding precise and meaningful descriptive terms.

Lastly, inspired by the AQoL, the PROMIS and the PWI, we suggest that the *social health domain* be described along two separate dimensions: *personal relationships*, i.e. the inner circle of the ‘nearest and dearest’ (family and friends), and; *social isolation*, i.e. participation in an outer circle reflecting the degree of community connectedness. Given that individuals differ in their preferred levels of social interactions, these two items should not be phrased in terms of quantities or frequencies, but rather by the extents to which people feel impaired to undertake their preferred levels of social activities.

In the context of the conceptual map, the suggested new descriptive system would contain: three symptom/impairment/cause indicators (pain, sleep, depression); three functioning/effect indicators (mobility, self-care, vitality), and; two ‘effect of effect’ indicators (personal relationships, social isolation).

## Discussion

The aim of this paper was first to develop a conceptual map to better understand in which ways the descriptive systems of five HSU-instruments differ. Previous comparisons of domain contents in HSU-instruments’ [1, 3] operate with a simplistic physical versus psycho-social dichotomy. An important contribution from the current paper lies in the extended conceptual analysis. Inspired by two models; the WHO-endorsed International Classification of Functioning, Disability, and Health (ICF), and the Wilson and Cleary model, our proposed framework was built around four key distinctions, which emerged more like continua than dichotomies: (1) cause versus effect; (2) specific versus abstract; (3) physical versus psychological, and; (4) subjective vs objective. Based on these continua, a visual framework was drawn, in which we located each of the five instruments, thereby illustrating their similarities and contrasts.

By mapping each HSU-instruments onto the same visual framework, their contrasts and similarities become apparent. In addition to visualising the differences in which dimensions that are included, the mapping exercise shows variations in how some dimensions are operationalised in terms of the number of items included and the wording or concepts used. Moreover, the conceptual map demonstrates the varying extent to which each instrument moves outside the traditional biomedical focus of physical health, to also incorporate indicators of mental health and social well-being.

The EQ-5D, which is the most widely applied instrument, is also the shortest. The conceptual map (Fig. 4) reveals that this instrument does not cover much of psychological/mental health, and it does not pick up on social aspects of health. However, in the development of the descriptive system, *Usual activities* was originally split in two: *Main activities* (e.g. work, study, housework) and *Family and leisure activities* [28], the latter brings connotation to social functioning. The AQoL contrasts the EQ-5D, in that it covers several aspects of mental and social health. A further contrast is that the AQoL is the longest instrument, and rarely applied, at least outside of Australia.

Following on from this conceptual map, the second aim of the paper was to draw lessons on what should be included in a new and improved HSU-instrument. In doing so, we started by extracting some common denominators that would be strong contenders for inclusion. Furthermore, acknowledging the increasing policy concern on the psycho-social aspects of health, we closely explored the only HSU-instrument (AQoL) that contains wide sets of items on mental health and social relationships. In addition, neighbouring instruments such as the PWI and the most recent PROMIS were explored. Finally, brevity was a critical concern.

The suggested new instrument leans towards the EQ-5D in terms of brevity and structure, and towards the AQoL by including key items on mental and social health. Much research remains on: (i) inquiring into the empirical support for the suggested dimensions based on existing data sets; (ii) how exactly the chosen dimensions should be phrased in easily understandable and meaningful wording; (iii) whether phrasing should depict the level of severity within the dimension alone or the *impact* the dimension has on functioning or general life domains; (iv) deciding on the number of levels that each dimension should be described, and; (v) which method(s) should be applied to develop preference based value sets.

In this paper, the choice of dimensions for a suggested *new* instrument was based on a theoretical investigation into *existing* instruments. Clearly, empirical research is required to decide whether our suggested eight (or nine) dimensions happen to be the most important ones. Hopefully, the results from a large research project currently carried out at the University of Sheffield (https://scharr.dept.shef.ac.uk/e-qaly/) may help provide an answer. Based on semi-structured interviews in six countries, the ‘Extending the QALY’ project seeks to identify candidate items for a new (extended) instrument from a list of 97 potential items. It remains to be seen where these candidate items will be located in our conceptual map, and the extent to which they will align with the key dimensions in existing instruments.

The choice of which instrument is used, whether it be an existing instrument, or a new or revised instrument, will ultimately be up to the users and their purpose for measuring HRQoL. However, this conceptual map provides a powerful tool for users of these instruments to better understand which descriptive system best aligns with what it is they are trying to assess. In addition, it provides a visual framework to which other existing HRQoL instruments can be mapped, whether generic or disease-specific, to allow users to ensure their operationalisation of HRQoL matches what it is that they are fundamentally aiming to capture.
